# Clinical outcomes of etoposide and cytarabine as consolidation in elderly patients with primary CNS lymphoma

**DOI:** 10.1093/oncolo/oyae059

**Published:** 2024-04-06

**Authors:** Yu Ri Kim, Hyunsoo Cho, Soo-Jeong Kim, Haerim Chung, Hye Won Kook, Ji Eun Jang, June-Won Cheong, Jin Seok Kim

**Affiliations:** Division of Hematology, Department of Internal Medicine, Gangnam Severance Hospital, Yonsei University College of Medicine, Seoul, South Korea; Division of Hematology, Department of Internal Medicine, Severance Hospital, Yonsei University College of Medicine, Seoul, South Korea; Division of Hemato-Oncology, Department of Internal Medicine, Yongin Severance Hospital, Yonsei University College of Medicine, Yongin, South Korea; Division of Hematology, Department of Internal Medicine, Severance Hospital, Yonsei University College of Medicine, Seoul, South Korea; Division of Hematology, Department of Internal Medicine, Severance Hospital, Yonsei University College of Medicine, Seoul, South Korea; Division of Hematology, Department of Internal Medicine, Severance Hospital, Yonsei University College of Medicine, Seoul, South Korea; Division of Hematology, Department of Internal Medicine, Severance Hospital, Yonsei University College of Medicine, Seoul, South Korea; Division of Hematology, Department of Internal Medicine, Severance Hospital, Yonsei University College of Medicine, Seoul, South Korea

**Keywords:** primary CNS lymphoma, consolidation, etoposide, cytarabine

## Abstract

**Background:**

A consolidation strategy has not been established for transplant-ineligible elderly patients with primary central nervous system lymphoma (PCNSL). In this study, we aimed to retrospectively evaluate the clinical outcomes of etoposide and cytarabine (EA) as consolidation chemotherapy for transplant-ineligible patients with PCNSL following high-dose methotrexate (MTX)-based induction chemotherapy.

**Materials and Methods:**

Between 2015 and 2021, newly diagnosed transplant-ineligible patients with PCNSL with diffuse large B-cell lymphoma were consecutively enrolled. All enrolled patients were over 60 years old and received EA consolidation after achieving a complete or partial response following induction chemotherapy.

**Results:**

Of the 85 patients who achieved a complete or partial response to MTX-based induction chemotherapy, 51 received EA consolidation chemotherapy. Among the 25 (49.0%, 25/51) patients in partial remission before EA consolidation, 56% (*n* = 14) achieved complete remission after EA consolidation. The median overall survival and progression-free survival were 43 and 13 months, respectively. Hematological toxicities were most common, and all patients experienced grade 4 neutropenia and thrombocytopenia. Forty-eight patients experienced febrile neutropenia during consolidation chemotherapy, and 4 patients died owing to treatment-related complications.

**Conclusion:**

EA consolidation chemotherapy for transplant-ineligible, elderly patients with PCNSL improved response rates but showed a high relapse rate and short progression-free survival. The incidences of treatment-related mortality caused by hematologic toxicities and severe infections were very high, even after dose modification. Therefore, the use of EA consolidation should be reconsidered in elderly patients with PCNSL.

## Teaser

This article evaluates the clinical outcomes of etoposide and cytarabine consolidation chemotherapy following high-dose methotrexate-based induction chemotherapy in transplant-ineligible patients aged >60 years with primary central nervous system lymphoma.

## Graphical Abstract

**Graphical Abstract:**
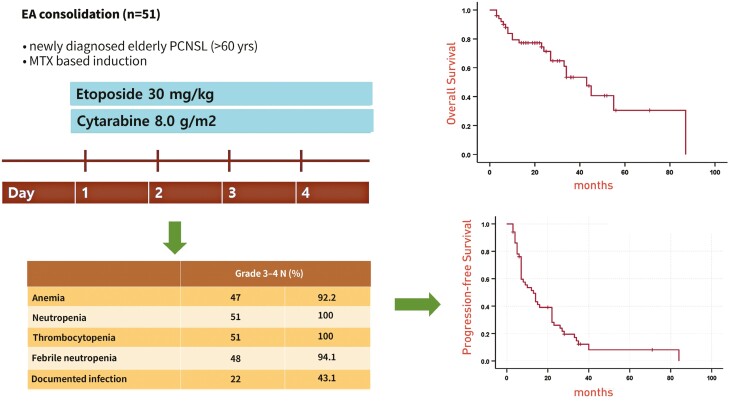

**Implications for Practice**

High-dose chemotherapy followed by autologous stem cell support is an effective consolidation strategy for young patients with primary central nervous system lymphoma (PCNSL). However, no standard consolidation method has been established for transplant-ineligible, elderly patients. Etoposide and cytarabine (EA) consolidation for transplant-ineligible elderly patients elicited a high complete response rate; however, the relapse rate was high and the progression-free survival was short. The incidences of hematologic toxicities and severe infections were very high, which eventually led to treatment-related mortality even after the dose modification. Therefore, the use of the EA regimen as consolidation should be reconsidered for use in elderly patients with PCNSL.

## Introduction

The incidence of primary central nervous system lymphoma (PCNSL) has increased over time, with the highest incidence rate in patients ≥75 years old.^1,2^ Although the clinical outcomes of PCNSL have improved since the introduction of high-dose methotrexate (MTX)-based induction chemotherapy, the survival of elderly patients has not dramatically improved.^2,3^ MTX-based induction chemotherapy elicits a considerably high response rate; however, most patients eventually experience relapses. Therefore, intensive consolidation therapy should be administered after achieving an appropriate response following induction chemotherapy.^2^ Myeloablative consolidation with high-dose chemotherapy followed by autologous hematopoietic stem cell transplantation (ASCT) is a preferred consolidation strategy for reducing recurrence and improving survival; however, this strategy is only applicable to young and fit patients.^4-6^ Because most elderly patients with PCNSL cannot tolerate ASCT, various efforts have been undertaken to develop appropriate consolidative strategies for elderly patients.^1,7^ Although radiation therapy remains a widely used consolidation strategy for disease control, there are significant concerns such as delayed neurocognitive toxicity presenting as dementia, gait ataxia, and urinary incontinence, particularly in elderly patients.^8-11^ Some studies have attempted to minimize delayed neurotoxicity by omitting whole-brain radiotherapy (WBRT) or reducing the radiation dose; nevertheless, long-term follow-up data and validation for a large population are required.^12-15^ In addition to delayed neurotoxicity, WBRT has some limitations for controlling disseminated lymphoma outside the radiation field.^16^

Other combination chemotherapeutic agents as consolidation, with mechanisms of action different from MTX, have been studied to avoid neurotoxicity from WBRT in transplant-ineligible patients.^8^ Consolidation of etoposide and cytarabine (EA) is one of the recommended regimens for consolidation therapy in patients with PCNSL. However, the consolidative role of EA chemotherapy for elderly patients has been minimally explored.^15,17,18^ A Cancer and Leukemia Group B (CALGB) 50202 trial suggested that EA consolidation was an effective, well-tolerated regimen and that the progression-free survival (PFS) among elderly patients was comparable to that among younger patients.^15^ Two other real-world studies on EA consolidation have also proven its efficacy. However, many enrolled patients experienced a higher incidence of toxicities. Furthermore, significant discrepancies exist between real-world data and recommendations based on clinical trials.^17,18^ Because previous studies on EA consolidation have been conducted in patients of all ages, the number of enrolled elderly transplant-ineligible patients was very small. Although current treatment guidelines recommend cytarabine-based consolidation chemotherapy for transplant-ineligible patients, the rationale for EA consolidation chemotherapy in elderly transplant-ineligible patients with PCNSL remains insufficient.^19^ Herein, we evaluated the clinical outcomes of EA consolidation chemotherapy following high-dose MTX-based induction chemotherapy in transplant-ineligible patients with PCNSL >60 years old.

## Materials and methods

### Patients

Between 2015 and 2021, newly diagnosed transplant-ineligible patients with PCNSL with diffuse large B-cell lymphoma were enrolled consecutively. The patients were registered from a prospective cohort at Severance Hospital (Yonsei University College of Medicine, Seoul, Republic of Korea; IRB 4-2014-0236, ClinicalTrials.gov ID: NCT02330718). Only patients > 60 years old who were immune competent were analyzed. Patients who did not receive at least one cycle of high-dose MTX-based chemotherapy or who did not respond to induction chemotherapy were also excluded. All enrolled patients received EA consolidation therapy after achieving a complete response (CR) or partial response (PR) after induction chemotherapy. Memorial Sloan-Kettering Cancer Center (MSKCC) and International Extranodal Lymphoma Study Group (IELSG) scores were calculated as previously described.^20,21^ This study was approved by the institutional review board of Severance Hospital.

### Chemotherapy

Induction chemotherapy was performed using one of the following regimens: MTX, vincristine, and dexamethasone (MVD regimen; MTX 3.5 g/m^2^ intravenously [i.v.] on day 1, vincristine 1.4 mg/m^2^ i.v. [maximum dose, 2 mg] on day 1, and dexamethasone 40 mg i.v. on days 1-5); MTX, vincristine, dexamethasone, and procarbazine (MVP regimen; MTX 3.5 g/m^2^ i.v. on day 1, vincristine 1.4 mg/m^2^ i.v. [maximum dose, 2 mg] on day 1, dexamethasone 40 mg i.v. on days 1-5, and procarbazine 100 mg/m^2^ on days 1-7 during odd cycles); and MTX, cytarabine, and thiotepa (MCT regimen; MTX 3.5 g/m^2^ i.v. on day 1, cytarabine 2 g/m^2^ twice daily i.v. on days 2 and 3, and thiotepa 30 mg/m^2^ on day 4). An etoposide and cytarabine consolidation regimen was administered at a total dose of 40 mg/kg (i.v. infused over 96 hours) and 16 g/m^2^ (2 g/m^2^ over 2 hours every 12 hours on days 1-4). Initially, etoposide 40 mg/kg was administered to 2 patients; however, they experienced septic shock during the neutropenic period (grade 4 sepsis). We then revised the institutional protocol and reduced the dose of etoposide by 25% (30 mg/kg) for a continuous period of 96 hours on days 1-4, and cytarabine 1 g/m^2^ was administered every 12 hours on days 1-4 (a total dose of 8 g/m^2^). All patients received antibacterial and antifungal prophylaxis with ciprofloxacin and fluconazole, and granulocyte-colony stimulating factor (G-CSF) was administered until neutrophil recovery.

### Response and toxicity assessment

The response was assessed using magnetic resonance imaging (MRI) based on the International PCNSL Collaborative Group response criteria.^22^ Induction treatment response was assessed by MRI after the second and fourth cycles of induction chemotherapy. The response was evaluated after the completion of EA consolidation chemotherapy, every 3 months for 2 years, and then every 6 months for 3 years. Toxicities were graded using the National Cancer Institute Common Toxicity Criteria for Adverse Events (version 4.0).

### Statistical analysis

Overall survival (OS) was defined as the duration from the date of diagnosis to the date of death from any cause. PFS was defined as the time from the date of diagnosis to progression, relapse, or death from any cause. Survival analyses were estimated using the Kaplan-Meier method. All reported *P-*values were 2-sided, and a *P*-value < .05 was considered statistically significant for all analyses. All statistical analyses were performed using SPSS Statistics for Windows, version 23.0 (IBM Corp., Armonk, NY, USA).

## Results

### Patients’ characteristics

**Figure 1.**
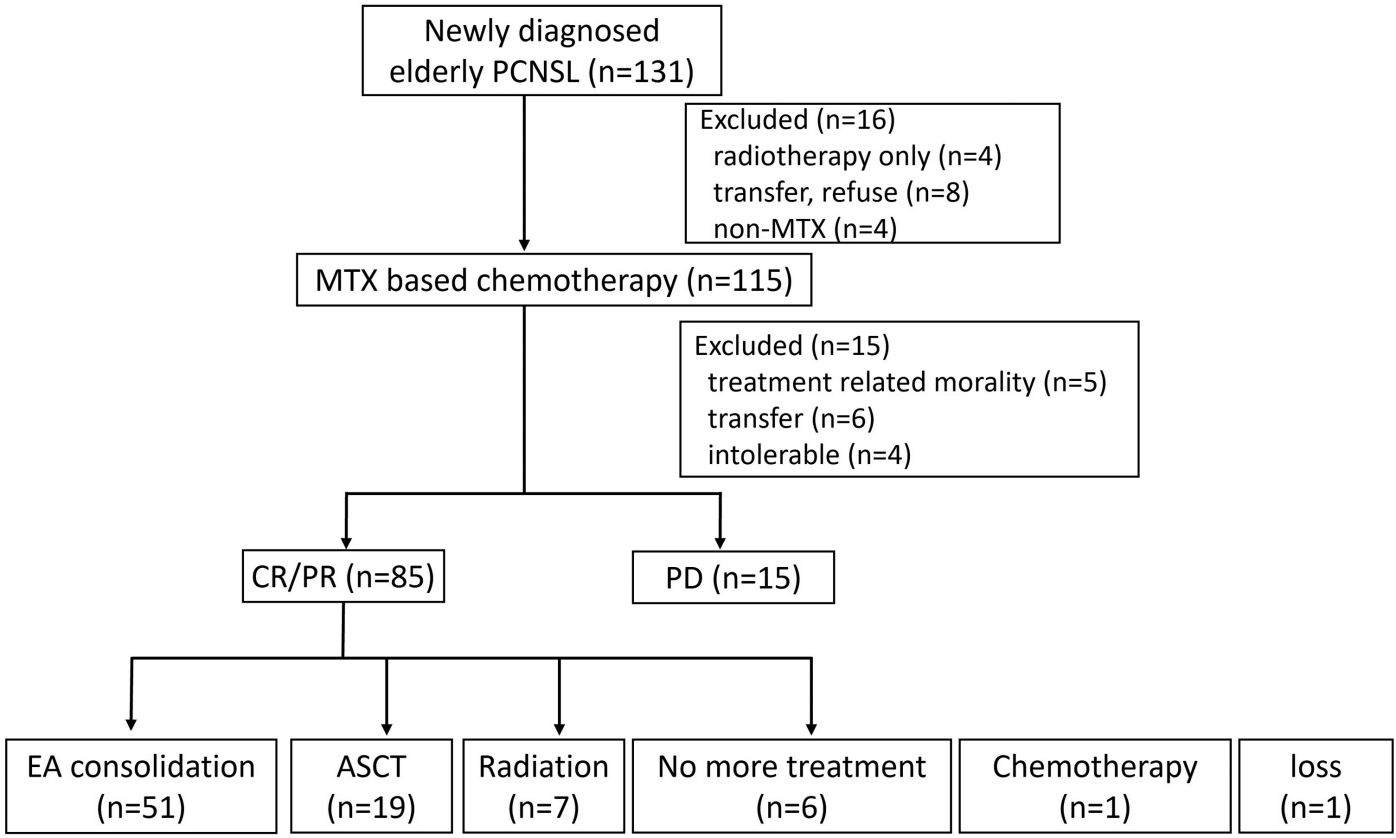
Study diagram. Abbreviations: ASCT, autologous hematopoietic stem cell transplantation; CR, complete response; PD, progressive disease; PR, partial response; RT, radiotherapy.

During the study period, 131 patients over 60 years of age were diagnosed with PCNSL at our institution. A total of 115 patients were treated with high-dose MTX-based induction chemotherapy. Among them, treatment responses were accessible in 100 (76.3%) patients, including patients with disease progression (*n* = 15, 15.0%) and CR or PR (*n* = 85, 85.0%). Among the 85 patients who achieved CR or PR after high-dose MTX-based induction chemotherapy, 19 underwent ASCT, 7 received radiotherapy, 6 did not receive further treatment, and 51 received EA consolidation. Finally, 51 patients who received EA consolidation treatment were included in this study. The study diagram is shown in Figure 1.

The baseline characteristics of the 51 patients who received EA consolidation are presented in Table 1. The median age was 71 (range: 61-79) years and there were 22 male patients. Of the 51 patients, 47 (92.1%) received MVD and 4 (4.9%) received MCT induction chemotherapy. The pathologic diagnosis of all patients was diffuse large B-cell lymphoma. Among the 48 available cases, 37 (77.1%) were classified as nongerminal center B-cell subtype, 11 (22.9%) as germinal center B-cell subtype, 6 (12.5%) as double-expressor lymphoma, and 42 (87.5%) as non-double-expressor lymphoma. According to fluorescence in situ hybridization analysis, 3 patients had *BCL6* translocation, and 46 patients showed no translocation between *MYC* and *BCL*2 and/or *BCL*6. Furthermore, 39 (76.5%) patients belonged to MSKCC risk group 2, and 12 (23.5%) belonged to MSKCC risk group 3. Forty-nine (96.0%) patients were available for IELSG risk assessment, and 48 (97.9%) had IELSG risk scores ≥ 2 (Table 1).

**Table 1.** Baseline characteristics.

Characteristics	EA consolidation (51 patients) *N* (%)
Age (years), median (range)	71 (61-79)
Males	22 (43.1)
ECOG 2-4	12 (23.5)
Elevated LDH	29 (56.9)
Deep brain lesion	30 (58.8)
Elevated CSF protein	41/49 (83.7)
Non-GCB subtype	37/48 (77.1)
MSKCC risk group	
2	39 (76.5)
3	12 (23.5)
IELSG risk group	
0-1	1/49 (2.0)
2-3	29/49 (59.2)
4-5	19/49 (38.8)

Abbreviations: CSF, cerebrospinal fluid; EA, etoposide and cytarabine; ECOG, Eastern Cooperative Oncology Group; GCB, germinal center B-cell; IELSG, International Extranodal Lymphoma Study Group; LDH, lactate dehydrogenase; MSKCC, Memorial Sloan-Kettering Cancer Center.

### Response and survival

All 51 patients in this study received at least 4 cycles of high-dose MTX-based combination chemotherapy, and treatment response was available. Twenty-six (51.0%) patients received EA consolidation in the CR state, and 25 in the PR state. Among the 25 (49.0%, 25/51) patients with PR before EA consolidation, 56% (14/25) eventually achieved CR after EA consolidation. Response evaluation after EA consolidation was assessable in 47 patients, with 37 (78.7%) achieving CR, 8 (17.0%) achieving PR, and the remaining 2 (4.25%) experiencing disease progression. Among these 8 patients with PR, 5 achieved CR following radiotherapy after EA consolidation, 1 received additional salvage chemotherapy, and 2 did not receive any further treatment because of poor performance status. Among the 37 patients who achieved CR following EA consolidation, 6 (16.2%) maintained the response, whereas 31 (83.7%) patients experienced relapses. The median time to relapse was 236 (range: 47-2471) days.

**Figure 2.**
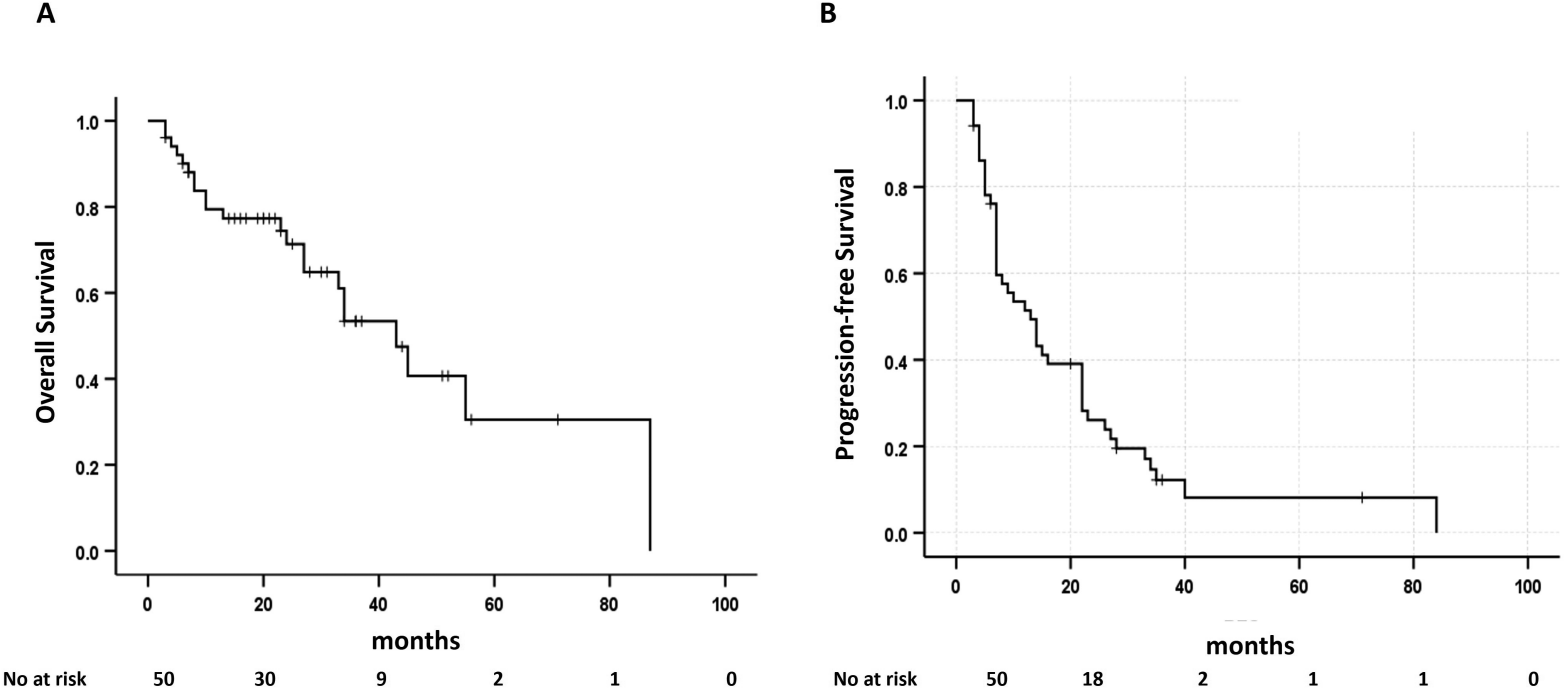
Overall survival (A) and progression-free survival (B) among patients who received etoposide and cytarabine (EA) consolidation.

The median follow-up period was 23 (range: 3-87) months for all 51 patients who received EA consolidation. Of the 51 patients, 6 (11.7%) were alive with disease-free status, 4 (7.8%) died due to treatment-related complications, and 31 (60.7%) experienced relapse after EA consolidation chemotherapy. Among the 31 relapsed patients, 4 achieved a CR again after salvage treatment, 14 patients did not achieve an appropriate response after salvage treatment or refused further treatment, and 13 patients died from relapse. The 2-year OS and PFS rates for the EA consolidation group were 71.7% and 26.1%, respectively (Figure 2).

### Toxicity

Most patients experienced grade 3-4 hematologic toxicities. All patients experienced grade 4 neutropenia and thrombocytopenia. The median period of neutropenia (absolute neutrophil count < 500/μL) was 10 (range: 6-20) days, and the median duration of hospital stay was 24 (range: 17-92) days for EA consolidation. Forty-eight (94.1%) patients experienced febrile neutropenia, and 4 (7.8%) died due to grade 5 febrile neutropenia. A total of 22 patients had documented infections caused by infectious organisms, such as vancomycin-resistant enterococci (1 in urine and 3 in blood), methicillin-resistant *Staphylococcus aureus* (1 in blood), *Klebsiella pneumoniae* (3 in blood and 3 in urine), *Escherichia coli* (1 in urine and 4 in blood), Gram-positive cocci (5 in blood), *Candida* (1 in blood), and *Pseudomonas* (1 in blood and 1 in sputum). The toxicity grades according to the National Cancer Institute Common Toxicity Criteria for Adverse Events are presented in Table 2.

**Table 2.** Common toxicities by grade during EA consolidation (*N* = 51).

Adverse events	Grades 1-2 *N* (%)		Grades 3-4 *N* (%)	
Anemia	4	7.8	47	92.2
Neutropenia	0	0	51	100
Thrombocytopenia	0	0	51	100
Diarrhea	22	43.1	0	0
Mucositis	16	31.3	3	5.9
Nausea	23	45.1	2	3.9
Febrile neutropenia	0	0	48	94.1
Documented infection	0	0	22	43.1
Elevated liver enzyme	13	25.5	2	3.9
Elevated creatinine	6	11.7	1	2.0

Abbreviation: EA, etoposide and cytarabine.

## Discussion

This study analyzed the efficacy and safety of EA consolidation in transplant-ineligible patients with PCNSL who responded to high-dose MTX-based induction chemotherapy. Although CR rates can be improved from 50% to 70% after additional EA consolidation therapy, a relapse rate of approximately 76% and very high rates of treatment-related hematologic toxicities and infections warrant further improvements, as this is a formally recommended consolidation strategy in elderly patients with PCNSL.

The induction and consolidation phases are the 2 main components of the therapeutic strategy for PCNSL. Although PCNSL is sensitive to high-dose MTX-based induction chemotherapy, most patients who do not receive additional appropriate consolidation treatment eventually cannot maintain the response due to the high rate of relapse. ASCT has been implemented as an effective consolidation strategy for younger fit patients; nonetheless, no standard consolidation strategy has been established in elderly transplant-ineligible patients.^2^

High-dose cytarabine penetrates the blood-brain barrier, and cytarabine combined with MTX produces a better outcome than MTX alone.^23,24^ The addition of cytarabine (3 g/m^2^/day for 2 days) after WBRT as consolidation is associated with a high response and disease control with acceptable toxicities.^13,25,26^ Etoposide can also cross the blood-brain barrier and lower the risk of CNS events in nodal diffuse large B-cell lymphoma.^27^ EA combination therapy has already shown favorable outcomes in refractory or recurrent PCNSL.^28^ Therefore, the combination of etoposide and high-dose cytarabine may be considered an appropriate consolidation regimen after high-dose MTX-based induction chemotherapy. Although several previous studies on EA consolidation reported remarkable efficacy and tolerable safety (Table 3),^15,17,18^ they could not confirm the role of EA consolidation in elderly transplant-ineligible patients because of the small number of enrolled patients older than 60. Therefore, our real-world evidence of EA consolidation chemotherapy for elderly patients with PCNSL would be useful in developing appropriate consolidation guidelines for this population.

**Table 3.** Comparison of studies related to EA consolidation in newly diagnosed PCNSL patients.

Authors	No. of patients who received EA consolidation	Age (median, range)	OS, months (median)	PFS, months (median)	Toxicity	Comments
Wieduwilt et al	14 (≥60, *n* = 6)	61, 40-84	NR	NR	Neutropenic fever (82%) TRM (1 patient)	MTR (methotrexate, temozolomide, and rituximab) induction
Rubenstein et al	29	12-76	NR	28.0	Febrile neutropenia (16%) TRM (1 patient)	
Birsen et al	14	39-77	NR	NR	Neutropenic fever (82%) TRM (none)	
Current study	41	>60	43.0	14.0	Febrile neutropenia (89.4%) TRM (1 patient)	High-dose MTX-based induction

Abbreviations: EA, etoposide and cytarabine; MTX, methotrexate; NR, not reached; OS, overall survival; PCNSL, primary central nervous system lymphoma; PFS, progression-free survival; TRM, treatment-related mortality.

The 2-year PFS and OS rates were only 26.1% and 71.7%, respectively, which were inferior to those of 2 previous studies for EA consolidation that reported rates of 78% and 93%, and 83% and 92%, respectively.^17,18^ However, caution is warranted when interpreting this difference in dose intensity. Because the first 2 patients who received the same dose of EA consolidation (etoposide 40 mg/kg total dose and cytarabine 16 g/m^2^ total dose) as reported in a previous trial^15^ experienced grade 4 septic shock during the neutropenic period, the dose of EA consolidation was modified (etoposide 30 mg/kg total dose, cytarabine 8 g/m^2^ total dose) for further enrolled patients. Despite these dose reductions and careful supportive care such as antibacterial and antifungal prophylaxis and G-CSF support, treatment-related mortality occurred in 4 patients, and the incidences of hematologic toxicities and documented infections were very high. Because the previously proposed dose of EA consolidation was not targeted for elderly patients, this dose modification is considered appropriate, and further investigation should be conducted to identify the appropriate dose intensity of EA consolidation for elderly patients.

According to the CALGB 50202 trial, EA consolidation showed a high CR rate, favorable PFS/OS, and mild treatment-related toxicities with 16% neutropenic fever.^15^ The most important thing to note is that approximately 20% of patients who were unfit for EA consolidation were excluded. It is unlikely that a higher rate of toxicities related to EA consolidation was reported in the real-world data (Table 3).^15,17,18^ In our study, neutropenic fever and documented infections were reported in 94.1% and 43.1% of patients, respectively, comparable to the incidences of toxicities in previous real-world data. Because of the relatively high rate of hematologic toxicities and infections, hospitalization for managing these toxicities was required for all patients. Although treatment-related mortality was observed in 2 patients, our strategy of dose-modified EA consolidation with intensive supportive care would be feasible in elderly patients with PCNSL. Nevertheless, the relatively short PFS was still a challenge in routinely applying EA consolidation in elderly patients with PCNSL. Therefore, a more effective and safe treatment strategy other than EA consolidation chemotherapy should be investigated for elderly transplant-ineligible patients with PCNSL. Promising results of maintenance therapy with temozolomide, procarbazine, and lenalidomide for PCNSL have been reported.^29-31^ Considering the pathophysiology of PCNSL, Bruton’s tyrosine kinase inhibitors, such as ibrutinib, may also be good candidates for maintenance therapy in elderly patients with PCNSL.

This study had some limitations. We retrospectively conducted this study at a single center. However, considering the low incidence of PCNSL, the number of 51 elderly patients who received EA consolidation can be considered sufficient for evaluating the efficacy and safety of EA consolidation in elderly transplant-ineligible patients with PCNSL. After induction chemotherapy, we attempted to perform ASCT whenever possible in patients who were younger than 70 years old, and patients who were not appropriate for EA consolidation such as poor performance status or serious co-morbidities were treated with radiotherapy, which may have a potential selection bias. To minimize selection bias, we analyzed all enrolled patients with PCNSL who received EA consolidation from a prospective registry (ClinicalTrials.gov ID: NCT02330718).

## Conclusion

Although EA consolidation for transplant-ineligible elderly patients elicited a high CR rate, the incidences of hematologic toxicities and severe infections were very high, which eventually led to treatment-related mortality even after dose modification. Therefore, EA consolidation should be reconsidered for use in elderly patients with PCNSL. In addition, other therapeutic strategies such as maintenance therapy based on the pathophysiology of PCNSL are eagerly required in elderly patients with PCNSL because of the high rate of relapse and relatively short PFS even after EA consolidation chemotherapy.

## Data Availability

The data used in this study are available on request from the corresponding author (hemakim@yuhs.ac). The data will be provided after de-identification, in compliance with applicable privacy laws, data protection for consent, and anonymization.
